# The Integration of Artificial Intelligence Into Precision Medicine for Neuro-Oncology: Ethical, Clinical, and Nursing Implications in Immunotherapy Care

**DOI:** 10.7759/cureus.85024

**Published:** 2025-05-29

**Authors:** Victoria M Ajibade, Chidinma S Madu

**Affiliations:** 1 Pediatric Critical Care, Duke University Health System, Durham, USA; 2 Neuro-Oncology, Duke University Health System, Durham, USA

**Keywords:** immunotherapy, neuro-oncology, nursing, patient care, precision medicine, targeted therapy, technology integration

## Abstract

This paper explores how artificial intelligence (AI) is being woven into precision medicine for neuro-oncology, highlighting its ethical, clinical, and nursing implications in the realm of immunotherapy. With AI-powered diagnostics and predictive analytics, we’re seeing a boost in treatment accuracy, which paves the way for more personalized and effective care. On the clinical side, AI is fine-tuning targeted therapies, leading to better patient outcomes and less treatment-related toxicity. However, ethical concerns pop up around data privacy, algorithmic bias, and fair access to these AI-driven treatments. Nurses are at the forefront of tackling these issues, ensuring that care remains patient-centered, monitoring AI-assisted interventions, and grappling with ethical challenges. Their role in education and advocacy is crucial in connecting the dots between AI innovations and compassionate care. As AI continues to advance, it’s vital for different disciplines to work together to tap into its potential while maintaining ethical standards and enhancing care in neuro-oncology.

## Introduction and background

Neuro-oncology, a subspecialty of oncology, has been revolutionized with the advent of precision medicine, which has enabled healthcare professionals to create individualized treatment plans based on patients' molecular and genetic characteristics. This individualized care has enormous potential, as it allows physicians not only to understand the patient's biology of the disease better but also to select treatments that are more likely to yield positive outcomes. The shift toward precision medicine ensures that patients receive personalized plans, which enhance the efficacy of treatment and minimize side effects immensely, ultimately resulting in better clinical outcomes and quality of life. As individualized attention gains greater importance, the application of cutting-edge technologies, such as artificial intelligence (AI), has emerged as a cornerstone in enhancing the precision of diagnoses and treatment protocols in neuro-oncology. AI plays an integral role in propelling diagnostic accuracy and real-time decision-making in neuro-oncology treatment [1].

AI technologies in the form of machine learning algorithms have now been incorporated into healthcare practice, wherein they are useful tools for early tumor detection, cancer typing, and forecasting treatment outcomes. Such AI-driven advances enable clinicians to offer more precise and prompt interventions, thus improving overall patient outcomes. In addition, AI is instrumental in monitoring patient progress and adjusting treatments in real-time, so that care plans are constantly updated based on the latest data and trends encountered in the process of a patient's treatment [2]. As a result, AI not only improves the diagnostic role of neuro-oncology specialists but also the overall effectiveness and efficiency of care delivery. Though there are several benefits of integration with AI, the adoption of these technologies offers a complex dance of professional, clinical, and ethical issues, particularly in neuro-oncology nursing. Though AI potentially simplifies workflows, improves clinical outcomes, and enables more precise, tailored care, it raises questions of patients' autonomy, data privacy, and algorithm transparency.

## Review

The field of neuro-oncology has seen major progress with the integration of AI, especially within precision medicine. AI is transforming how we diagnose, treat, and monitor brain and central nervous system tumors by analyzing complex genetic, molecular, and clinical data with impressive accuracy. One key breakthrough is the development of personalized immunotherapies, which have greatly improved treatment outcomes. However, the rapid use of AI also raises ethical concerns, such as data privacy, algorithmic bias, and the importance of keeping human oversight in clinical decision-making. Nurses play a vital role in this evolving landscape by ensuring ethical use, educating patients, and helping maintain a balance between technology and compassionate care. Moving forward, collaboration among healthcare professionals, along with strong regulatory frameworks such as the Health Insurance Portability and Accountability Act (HIPAA) and the General Data Protection Regulation (GDPR), will be essential to ensure that AI enhances neuro-oncology care safely and equitably.

One of the most important ethical issues is ensuring the protection of sensitive patient information, with AI systems often requiring access to significant amounts of medical data for processing. Ensuring the confidentiality and security of such data is paramount to maintaining patient trust and regulatory compliance. In addition, AI application in clinical decision-making raises concerns regarding human supervision of automated systems, especially when algorithms issue suggestions that could impact treatment decisions. It is important to ensure that healthcare professionals maintain control of key decisions and that AI remains a supporting tool and not a replacement for human judgment [3].

Nurses, as integral members of the healthcare team, face both challenges and opportunities as AI becomes increasingly embedded in neuro-oncology care. Introduction of AI into practice requires new learning abilities and information, particularly using sophisticated AI technology in patient evaluation, care planning, and tracking outcomes. Nurses must not only be educated on the technical operation of these technologies but also about the ethics of such tools [4]. Situated at the forefront of care, nurses play a significant role in connecting insights gleaned from AI to patient-centered, compassionate care. Nurses must stand in for the needs and rights of patients, ensuring that AI technology is used to complement, and not replace, human touch and decision-making. Besides all this, the nurses have the responsibility of maintaining constant monitoring and evaluation of the safety and effectiveness of the applied AI systems in such a manner that proper adoption of technologies and prioritization of patient safety are ensured without a hitch. 

Ethical considerations of a multifaceted nature involve informed consent issues, bias within algorithms, as well as matters regarding possible dehumanization of care in practice. An overall practice of clearly educating patients on the use of AI in their treatment plan and offering them the choice of whether to consent to its utilization is an essential practice of maintaining ethical standards within the clinical environment.

Additionally, AI systems must be tested regularly for fairness and accuracy to avoid the reinforcement of biases that will negatively affect particular patient populations. Human judgment, accountability, and the preservation of empathetic care must remain in front of AI applications, so that technology is employed as an adjunct to, rather than a replacement for, human judgment and experience. This review aims to address the growing convergence of AI and precision medicine in neuro-oncology, addressing both the clinical and ethical concerns arising from the application of these technologies in patient care [5]. By examining the evolving roles of healthcare professionals, this paper illustrates how AI is revolutionizing clinical practice, enhancing diagnostic accuracy, and advancing personalized treatment. At the same time, it emphasizes the importance of developing solid ethical standards for the effective implementation of AI in neuro-oncology. The review demands ongoing professional training and education to equip health professionals with the competence and know-how required to comprehend the nuances of AI-based care while, at the same time, ensuring that patient well-being remains paramount. Ultimately, the use of AI in neuro-oncology can potentially revolutionize cancer therapy, but only if it reconciles technological innovation with maintaining empathetic, human-centered medicine [6].

AI-driven diagnostics and personalized treatment planning

Neuro-oncology is rapidly evolving with the integration of AI to improve diagnostic accuracy and develop personalized treatment approaches for patients. One of the most significant advancements in diagnostic precision has been the incorporation of AI-based technologies, which have transformed traditional methods of neuro-oncological disorder diagnosis. Historically, diagnoses relied on clinicians manually reviewing clinical images, pathology reports, and patient histories-processes that, while effective, are time-consuming and prone to human error [7]. With AI systems in place, advanced imaging analysis and radiomics now enhance tumor identification and classification capabilities, enabling faster and more accurate diagnoses. Machine learning algorithms are particularly adept at processing vast datasets and identifying patterns in medical images that may be invisible to the human eye. This ability facilitates earlier and more precise diagnostic outcomes, significantly improving the speed and accuracy of identifying tumors. Deep learning models trained on large databases excel at distinguishing tumor subtypes, which provides invaluable insight for making optimal treatment decisions [8]. These predictive algorithms not only enhance tumor identification but also forecast tumor growth, offering critical information that helps medical teams select the most appropriate treatment strategies. AI's utility extends beyond imaging to the analysis of genetic mutations in tumors. By leveraging AI-based algorithms, healthcare providers can identify actionable mutations that guide the selection of tailored therapies. For instance, AI models have proven particularly effective in detecting mutations that respond to therapies like epidermal growth factor receptor (EGFR) inhibitors, isocitrate dehydrogenase (IDH)-targeted therapies, and immune checkpoint inhibitors.

These advancements in genetic analysis have allowed for more precise treatment plans that increase the likelihood of successful outcomes. In clinical settings, AI-driven decision support systems are increasingly utilized to process genomic data in real time, allowing clinicians to quickly detect mutations and determine the most effective treatment options. These systems enable neuro-oncology specialists to accurately diagnose tumors and develop patient-specific treatment plans that incorporate genetic information, further personalizing the approach to care. Nurses, as essential members of the healthcare team, play a crucial role in the implementation of AI-assisted diagnostics and the creation of personalized treatment plans. While they may not be directly involved in the technological processes, their role in patient education is paramount. Nurses must explain AI-generated diagnostic results and treatment plans to patients, ensuring that individuals understand how these technologies enhance their care.

Furthermore, they must provide patients with comprehensive information regarding the risks and benefits of AI-based treatments through informed consent protocols. Nurses are also responsible for safeguarding patient privacy and addressing data security concerns as AI technologies become increasingly integrated into treatment planning. To effectively integrate AI into their practice, nurses must continually update their knowledge of emerging AI technologies through regular educational initiatives. By staying informed about AI advancements, nurses can help optimize the application of these technologies in neuro-oncology, ensuring that patient care remains personalized, ethical, and patient-centered. In conclusion, AI-driven diagnostics and personalized treatment planning have revolutionized neuro-oncology, enhancing diagnostic accuracy and enabling individualized therapies. Although nurses may perform a more indirect role in these processes, their contributions are crucial, particularly in educating patients and ensuring the ethical implementation of AI in clinical practice.

AI's role in enhancing patient education and decision-making

The way AI is being integrated into neuro-oncology is truly transforming how patients learn about their conditions and make decisions regarding their treatment. With real-time insights into treatment options and disease progression, AI tools like chatbots, virtual assistants, and decision-support systems are helping to close the knowledge gap between healthcare providers and patients. This ensures that individuals have a much clearer grasp of their health and the treatment plans available to them. These innovative tools use natural language processing (NLP) and machine learning algorithms to break down complex medical jargon into straightforward information, making it easier for patients and their caregivers to understand. One of the standout benefits of AI is its knack for simplifying complicated medical details, customizing explanations to match each patient’s understanding. AI-driven platforms can create personalized reports, visualize how tumors are progressing, and even simulate potential treatment outcomes, empowering patients to make well-informed decisions about their care. Take, for instance, AI imaging systems like IBM Watson Health, which can analyze MRI and CT scans to provide clear visuals of tumor growth, helping patients better understand their situation. This kind of transparency not only empowers patients but also fosters trust and engagement in their healthcare journey [9].

AI is stepping up the game when it comes to shared decision-making in healthcare. It provides clinicians with valuable, data-driven insights that they can easily share with patients clearly and understandably. By sifting through massive amounts of clinical data, AI can help predict how patients might respond to different treatments and what side effects they might experience. This collaboration allows doctors and patients to work together in choosing the best therapeutic options, including personalized immunotherapy. For example, platforms like Tempus (Tempus AI, Inc., Chicago, IL) use AI to analyze genetic and molecular profiles, recommending targeted immunotherapy treatments that are specifically tailored to a patient’s unique tumor characteristics. This approach not only aims to deliver the most effective treatment but also seeks to minimize any adverse effects [10].

Another great example is AI-powered chatbots, like Ada Health (Ada Health GmbH, Berlin, Germany), which help patients assess their symptoms and provide educational resources about their conditions. These chatbots let patients enter their symptoms and get initial insights into their health, guiding them to seek medical advice when needed. This proactive method boosts patient engagement and encourages early intervention, which can lead to better health outcomes. Additionally, AI-driven virtual reality (VR) and augmented reality (AR) applications are being utilized to educate patients about their treatment options. Companies such as Oncomfort (Switzerland) offer VR-based therapeutic sessions that allow patients to visualize their upcoming procedures, helping to ease anxiety and enhance their understanding of complex treatments like immunotherapy [11]. By immersing patients in an interactive learning experience, AI empowers them to feel more prepared and confident in making informed healthcare decisions.

While AI brings a lot to the table, we can't overlook the ethical issues that come with it. It's essential to make sure that patient autonomy and privacy are respected. We need to ensure that the recommendations made by AI are easy to understand, unbiased, and backed up by human judgment to keep patient trust intact. Nurses are vital in this process; they help turn AI insights into caring, patient-focused conversations, making sure individuals feel supported as they make decisions. They guide patients through AI-generated treatment options, answer any questions about therapies, and offer emotional support throughout the decision-making journey.

Ethical considerations in AI-driven precision medicine

The application of AI in neuro-oncology is a game-changing development in the precision medicine of brain cancer diagnosis and treatment. AI tools allow clinicians to process and interpret vast amounts of medical information, including medical images, genomic information, and patient records, far more efficiently than traditional methods. This capability enables doctors to gain more insight into a patient's status, identify patterns not necessarily discernible to the human eye, and create very personalized treatment regimens that meet the specific needs of each patient [12]. By leveraging AI, clinicians can make more informed decisions about the most effective course of treatment, resulting in improved outcomes and a more targeted approach to care. However, as with every technological advancement, the integration of AI into clinical practice also ushers in significant ethical concerns that need to be carefully explored and addressed. One of the most significant challenges is how to maintain patients' rights within the AI-driven decision-making process. Patients, for example, must be adequately informed regarding how AI is used in their care and the possible risks and benefits of AI-driven intervention.

Informed consent under such circumstances then becomes all the more complex since, though patients might not fully understand how the AI systems operate, this renders autonomy and control of decision-making capacities suspect. As AI plays a larger role in treatment planning, patients may question how much these systems should be allowed to control their care. In addition, the ethical use of AI in neuro-oncology requires that patient safety is always the top priority. AI systems are robust but imperfect. Errors in data interpretation, algorithmic biases, or predictive modeling inaccuracies can result in incorrect diagnoses or inappropriate treatment planning that can be detrimental to patients [13]. Healthcare professionals must have rigorous oversight over AI-generated recommendations and verify them against long-established clinical standards and individual patient needs. This level of supervision is necessary to prevent harm and ensure that AI is a positive aid to enhancing, rather than detracting from, patient care. Moreover, the rapid development of AI technologies in healthcare has raised concerns about data collection, storage, and exchange. AI tools rely heavily on patient data, including sensitive information like genetic information, medical histories, and imaging reports. 

This has significant implications for data privacy and security. If not adequately protected, such data can be at risk of unauthorized access, misuse, or theft, undermining patient confidentiality and trust in the health system. Ensuring that AI systems are compliant with stringent data protection regulations, such as HIPAA in the United States or GDPR in Europe, is imperative in safeguarding patient information and maintaining the sanctity of the healthcare environment [14]. As AI continues to evolve in neuro-oncology, such ethical dilemmas must be addressed by thoughtful policies, transparent practices, and ongoing dialogue among clinicians, ethicists, and patients. The goal is to harness the vast potential of AI to improve patient outcomes without compromising the fundamental principles of medical ethics, including respect for patient autonomy, preservation of privacy, and provision of safe, effective care. 

By carefully considering these ethical factors, the healthcare industry can introduce AI in a way that improves the accuracy and customization of neuro-oncological care without compromising patient trust or well-being like.

Data Privacy and Security

Neuro-oncology AI platforms must have access to large, sensitive data to function effectively. These datasets typically consist of sensitive patient information in the form of genetic data, medical images, and health records. The security and confidentiality of such information must be ensured. Breach of patient confidentiality by unauthorized individuals can result in identity theft, discrimination, or compromise of patient privacy. Therefore, there is a need to have robust security controls and adhere to privacy laws such as HIPAA in the United States and GDPR in Europe. These laws are enacted to protect patient data from misuse and ensure that AI systems are operating within legal and ethical boundaries. The ethical dilemma is how to balance the innovation and potential advantages of AI with strict patient data protection.

Algorithmic Bias and Fairness

AI systems are only as good as the data that they are trained on, and the other major ethical challenge of precision medicine with AI is algorithmic bias. If the training sets for AI systems are not generalizable to a large population of patients based on race, gender, and socioeconomic status, then the system will most likely not function well for underrepresented populations. This bias may lead to false diagnoses, discriminatory treatment recommendations, or suboptimal care for certain patient groups. To enhance fairness and equity in the provision of care, AI systems must be trained using representative and diverse datasets. This approach makes it possible for AI to give more accurate, unbiased, and equitable healthcare to all patients, regardless of their demographic background. Addressing this issue is imperative to prevent discrimination and guarantee that AI can be advantageous to all patients uniformly.

Human Monitoring and Accountability

Human monitoring remains essential despite the high capabilities of AI. AI is a method of enhancing rather than replacing the function of physicians and healthcare practitioners. Doctors and nurses must remain actively involved in the decision process, using AI-derived information within an expanded clinical judgment framework. Doctors, for their part, should carefully examine the recommendations made by AI programs and make sure that they are consistent with clinical best practices as well as with the specific needs of the patients. It is of the utmost importance that human professionals remain ultimately responsible for determining the direction of treatment, as that responsibility ensures the safety of the patient and serves to detect any potential flaws or bias in the AI system. AI can be a valuable assistant, but should never replace the responsibility of healthcare providers to make informed, ethical decisions.

The role of nurses in AI-integrated neuro-oncology care is vital and multifaceted, encompassing both clinical responsibilities and the management of emerging technologies that assist in patient care. As AI becomes more embedded in the diagnosis and treatment of brain cancer, nurses are positioned at the forefront of ensuring that these advancements are used ethically and effectively while maintaining patient-centered care. Their involvement goes beyond the traditional roles of providing physical care and extends into facilitating the integration of AI tools into clinical practice, ensuring that these technologies serve to enhance patient outcomes.

The role of nurses in AI-integrated neuro-oncology care

Nurses serve as crucial mediators between AI technologies and patients, helping to interpret AI-driven diagnostic results and treatment plans. In many cases, AI systems generate complex data that can be difficult for patients to understand. Nurses are essential in translating these results into terms that patients can comprehend, thus ensuring informed consent and promoting patient autonomy. They must educate patients about how AI is used in their care, the potential benefits, and the risks involved, empowering patients to make informed decisions about their treatment options. In this context, nurses play a central role in fostering trust between the patient and the healthcare system, particularly when AI systems are involved in decision-making processes. In addition to their educational role, nurses must also be advocates for patient safety in AI-integrated care. While AI has the potential to significantly enhance the accuracy and efficiency of diagnostics and treatment planning, there are inherent risks, such as data inaccuracies, biases in algorithms, or system errors [15]. Nurses must stay vigilant and ensure that AI-driven recommendations align with clinical guidelines and patient needs. Their expertise in patient care allows them to provide an essential layer of oversight, identifying discrepancies or concerns that may arise from the use of AI. This is particularly important in neuro-oncology, where patient conditions can be highly complex and require individualized, nuanced care.

Furthermore, nurses must stay up to date with the latest advancements in AI technology and how they can be applied to neuro-oncology care. Ongoing education and training in these technologies are essential for nurses to effectively use AI tools in their practice. They need to understand not only the technical aspects of AI applications but also the ethical implications, particularly related to patient privacy, data security, and the management of sensitive medical information. By acquiring these skills, nurses can ensure that AI is used responsibly and that it complements the compassionate, human-centered care they provide. As AI continues to evolve, nurses will also need to adapt their roles to include more active participation in the development and implementation of AI-driven solutions. 

They are uniquely positioned to contribute insights on the practical aspects of patient care that can inform the design of AI systems, ensuring that these tools are optimized to meet the real-world needs of patients. Nurses’ firsthand knowledge of patient experiences, coupled with their clinical expertise, can help shape AI applications that are more effective, intuitive, and aligned with patient care goals. Ultimately, the role of nurses in AI-integrated neuro-oncology care is essential in bridging the gap between technology and human touch. They ensure that while AI contributes to advancements in diagnosis and treatment, the heart of care remains grounded in empathy, communication, and patient advocacy. Nurses are key to maintaining the ethical application of AI technologies and ensuring that they serve to enhance, rather than replace, the personalized, compassionate care that is central to the practice of neuro-oncology [16].

Challenges and future directions

The advancements in neuro-oncology through AI enable the advancement of precision medicine. The complete realization of its advantages demands that we resolve multiple remaining obstacles [17]. AI difficulties in clinical practice stem from insufficient standardization of verification procedures for medical AI systems, combined with a lack of measurement protocols. Adequate regulatory procedures will guarantee both the safety and performance of AI tools for patient care [18]. The absence of proper standards threatens to create variable medical services, which might endanger patient safety. The implementation of established standards to evaluate and approve AI systems will enable medical staff to trustfully use AI while simultaneously protecting patient welfare. Modern healthcare demands nurse and professional healthcare worker training regarding the optimal utilization of these emerging technologies. A continuous training system should be created to train personnel about AI principles so they can understand its functions, together with decoding its recommendations, while building competent data-driven clinical choices [19]. The ability to handle AI tools properly comes second to building essential reasoning strengths needed for data-based medical choices in patient treatment.

Tagging AI with telemedicine creates revolutionary neuro-oncology care methods for patients living in areas without adequate medical services. Virtual platforms allow physicians to track patient symptoms in real time, along with providing specialists' consultations and enabling patients to get expert assistance through digital means without distant travel requirements [20]. Telemedicine services will become more effective with AI advancement, which can decrease specialized care challenges to provide neuro-oncology treatment to broader patient populations [21]. Research directed towards the future needs to enhance AI transparency because medical practitioners require a clear understanding of AI's decision-making approaches. The adoption of AI requires better ethical frameworks to determine its proper use in oncology care, exclusively for patient welfare. Strategies should be established to integrate AI technology smoothly within healthcare professionals' workflows, particularly nurses, to guarantee that AI boosts instead of hindering patient care delivery.

## Conclusions

The integration of AI into neuro-oncology represents a significant advancement in diagnosing, treating, and monitoring brain and central nervous system tumors. Through AI’s ability to process and interpret vast amounts of genetic, molecular, and clinical data, healthcare providers are now able to deliver more accurate diagnoses and create highly personalized treatment plans, such as tailored immunotherapies that significantly improve patient outcomes. However, as this technology becomes more embedded in clinical practice, it brings with it complex ethical concerns. Issues such as patient data privacy, potential biases in AI algorithms, and the risk of over-reliance on automated systems highlight the continued need for human oversight and ethical vigilance.

Nurses play a critical role in this evolving landscape - not only as caregivers but also as educators, advocates, and ethical stewards. They help patients understand AI-driven treatment options, monitor AI recommendations in real time, and ensure that the use of such technology aligns with principles of fairness, transparency, and patient-centered care. Looking ahead, the successful and responsible implementation of AI in neuro-oncology will depend on strong interdisciplinary collaboration, ongoing professional education, and the reinforcement of regulatory protections like HIPAA and GDPR. By maintaining a thoughtful balance between innovation and ethical responsibility, AI can be a powerful tool in advancing the quality and equity of neuro-oncological care.
